# Supplementary material for: Smartphone intervention to optimize medication-assisted treatment outcomes for opioid use disorder: study protocol for a randomized controlled trial
Source: Trials. 2023 Apr 4;24:255. doi: 10.1186/s13063-023-07213-3 (PMC10071730; doi:10.1186/s13063-023-07213-3)
Supplement: Supplementary file 1 — Additional file 1. [file 13063_2023_7213_MOESM1_ESM.pdf]

Study Title:	Neurocognitive Mechanisms Underlying Smartphone-Assisted Prevention of Relapse in Opioid Use Disorder
PI (researcher):	G. Andrew James, PhD
Institution:	University of Arkansas for Medical Sciences

Key Information for "Neurocognitive Mechanisms Underlying Smartphone-Assisted Prevention of Relapse in Opioid Use Disorder"
MAIN STUDY

This first part gives you key information to help you decide if you want to join the study. We will explain things in more detail later in this form.

We are asking if you want to volunteer for a research study about testing a smartphone app to reduce opioid misuse. By doing this study, we hope to learn if this smartphone app can improve treatment outcomes for opioid use disorder.

Please ask the research team if you have any questions about anything in this form. If you have questions later, contact the researcher in charge of the study. The contact information is given later on this form.

What will happen if I join the study?

If you join, your part in this research will last about 6 months.

During the study, we will assign you to a group.

- One group will use a smartphone app daily to record opioid craving and withdrawal symptoms. The app will also warn you if you are entering an area with high risk of relapse.
- The other group will not use the app.
- Both groups will be contacted every month to complete surveys about opioid misuse.
- Both groups will be invited to an optional MRI study to understand how the brain changes with recovery from addiction.

Do I have to join this study?

No. It is okay to say no. You will not lose any services, benefits, or rights you would normally have if you decide not to join. If you decide to take part in the study, it should be because you really want to volunteer.

What do I need to know to decide if I should join this study?

People decide to join studies for many reasons. Here are some of the main things you should think about before choosing to join this study.

Main reasons to join the study

- ✓ The smartphone app may help reduce relapse.
- ✓ The MRI sub-study may show how the brain changes during recovery, which may inform future clinical care.

Main reasons not to join the study

- ✓ You may feel uncomfortable completing the app daily ratings of craving and withdrawal symptoms, or may feel uncomfortable discussing opioid use in the monthly follow-up phone calls.
- ✓ One feature of the app will use your phone's GPS to detect if you are entering an area of high risk of relapse. You may have privacy concerns using this GPS feature.
- ✓ The optional MRI study uses a powerful magnet to detect brain activity, which may affect metal in your body (pacemakers, screws, plates) by heating, moving, or turning off.

These are just some of the reasons to help you decide if you want to join the study. We will explain more about the risks, benefits, and other options to joining the study later in this form.

Tell the study team if you decide that you do not want to be in the study. Remember, it is okay to say no. You can still get your medical care from UAMS and affiliated MOUD clinics if you are not in the study.

University of Arkansas for Medical Sciences Informed Consent Form

- **We are asking you to be in a research study. You do not have to join the study.**
- **You can still get your medical care from UAMS and affiliated MOUD clinics even if you are not in the study.**
- **Take as much time as you need to read this form and decide what is right for you.**

Why am I being asked to be in this research study?

- We want to learn more about using a smartphone app to reduce relapse in opioid misuse
- By doing this study, we hope to find out whether a smartphone app can improve treatment outcomes for patients receiving medication assisted treatment (MAT) for opioid use disorder (MOUD)
- We are asking people like you, who are receiving outpatient MAT through UAMS CAST or an affiliated MOUD clinic, to help us.
- 255 adults ages 18 and older will be part of this study.
- We will also invite participants to an optional MRI study. This informed consent form only discusses the main study. We have a separate informed consent form for the MRI study if you are interested.

What if I don't understand something?

- This form may have words you do not understand. If you would like, research staff will read it with you.
- You are free to ask questions at any time – before, during, or after you are in the study.
- Please ask as many questions as you would like before you decide if you want to be in this study. If you decide to take part in the study, it should be because you really want to volunteer.

What will happen if I say yes, I want to be in this study?

First, we will see if you qualify to be in the study. We will confirm that you are 18 or older, that you are enrolled in Phase 1 of MAT at UAMS CAST or an affiliated clinic, and that you have completed your intake visit and at least one weekly individual therapy session.

If you qualify, we will do these things:

- Give you a form with questions about your demographic information and drug use like your age, sex, race/ethnicity, education, and history of drug use.
- We will help you to fill a calendar describing the number of days you used drugs over the past month, and the types of drugs used each day. You will complete this calendar today in person, and once a month by phone for the next 6 months.
- We will give you a form about any pain you are experiencing. You will complete this pain form today, and once a month by phone for the next 6 months.
- We will give you a form about your income. You will complete this form today and over the phone in 6 months.
- We can help you by reading the questions out loud and fill out the form with you, if you like.
- You do not have to answer any questions you do not want to answer.
- Today, we will assign to one of two groups. One group will use a smartphone app daily to record daily opioid craving, withdrawal severity, mood, and use of alcohol or marijuana. The other group will not use the smartphone app. Your assignment to the group will be random, like flipping a coin.
- If you are in the smartphone group, we will install the app on your phone as show you how to use it.
- If you are in the smartphone group, you must agree to keep your phone locked with a passcode.
- If you are in the smartphone group, we will also ask you to give at least three locations where you are at high risk for relapse. These could be places where you buy opioids or where you have high craving for opioids. We will program these locations into our computer server. Our server will then detect if your phone GPS enters one of these areas. If your phone stays in one of these high-risk areas for longer than 5 minutes, the app will ask you to rate your current level of craving. The app may then ask you to reach out to a friend for help in getting out of that area before you relapse.
- Today's visit will last about an hour. Completing the smartphone ratings will take about 2 minutes each day. The follow-up monthly phone call will take about 10 minutes each.
- Throughout the study, we will record the weekly urinalysis drug test results from your CAST medical record.

How long will I be in this study?

You will be in the study for 6 months. It will include today's visit and 6 monthly follow-up phone calls.

What if I say no, I do not want to be in this study?

- Nothing bad will happen because of what you decide.
- You can still get medical care at UAMS and affiliated clinics.

What happens if I say yes but change my mind later?

- You can stop being in the study at any time.
- Nothing bad will happen because you change your mind and leave the study.
- You can still get medical care at UAMS and affiliated clinics.
- If you decide to stop being in the study, please inform the study team so that we will not continue to call you with the monthly follow up visits.
- Once you leave the study, you will not be able to re-enter the study later.

Will it cost me anything to be in the study?

The study will not cost you anything. You or your insurance company will be responsible for the costs of your regular medical care, as usual.

Will I be paid for being in the study?

Yes. After enrolling in the study today, we will give you your choice of $60 or a new smartphone worth $60. This is to compensate you for your time. After each of the monthly phone calls, we will send you a check for $10, or $60 total. If you complete all 6 of the monthly phone calls, we will send you a completion bonus of $50. In other words, you could receive up to $170 for completing all study activities.

All payments will be made by check at the end of each study visit. We cannot replace any lost or stolen checks.

If you get more than $600 in one year (January-December) from UAMS, we may send you a tax form if the law requires it.

Will being in this study help me in any way?

Being in the study may or may not help you, personally. But even if it does not help you, it may help people with opioid use disorder in the future. What we learn may help in the following ways:

- Using the smartphone app daily may prevent relapse of opioid misuse.
- Even if the smartphone app does not help, your feedback and the information we gain may help us improve the app for others.

What are the risks of being in this study?

The risk of joining this study are:

Study Privacy. There is the risk that someone could find out that you were in the study and learn something about you that you do not want others to know. We will do our best to protect your privacy, as explained in more detail later in this form.

Smartphone Privacy. There is a risk that the data we collect from your smartphone may have information that you do not want others to know, such as when you enter an area of high risk for relapse. We will do our best to protect your smartphone privacy, as explained below.

Legal Concerns. You may share with us details about illegal activities such as opioid misuse. We have legal protections to ensure your confidentiality, as explained below.

Mood. Being in the study may affect your mood or cause stress, such as:

- The questions asked may make you sad or upset.
- Thinking about your craving and withdrawal may make them worse.

What if I get sick or hurt while I am in this study*?*

- If you get hurt or sick when you are here for the study, we will help you get the care you need. This may include first aid, emergency care, and any follow-up care you need.
- If you are not here and get hurt or sick, and you think it is because of the study, do these things:
  - ✓ call your doctor – or if an emergency, call 911
  - ✓ give your doctor or ER staff
    - o the name of this study – Neurocognitive Mechanisms Underlying Smartphone-Assisted Prevention of Relapse in Opioid Use Disorder
    - o the name of the head researcher for this study – Andrew James, PhD
    - o a copy of this form if you have it

- ✓ call our study center at 501-420-2653. We will call you back during office hours.

- This treatment will be billed to you or your insurance company. No other form of payment is available.

Reminder: You do not give up any of your legal rights by agreeing to be in this study or by signing this form.

What are the alternatives to being in this study?

You do not have to be in this study. If you decide not to join this study, you still receive treatment at UAMS and affiliated clinics as usual.

Can I be taken out of the study even if I want to continue?

Yes, the study doctor (or head researcher) can take you out of the study if:

- You do not follow study instructions.
- You are not truthful about your health or medical history
- The study doctor (Dr. James) determines it is not in your best interest to continue.
- The study is stopped for any reason.

What information will be collected about me in the study?

During the study, we will need to learn private things about you, including

- General contact and background information about you, such as your name, address, telephone number, and other demographic information.
- Medical information about you, such as your weekly urinalysis drug test results
- Personal information, such as use of drugs and alcohol each month and if you experience chronic pain
- Smartphone information, such as places you have visited and smartphone ratings of daily opioid craving, opioid withdrawal, mood, and drug and alcohol use

Who will see this information? How will you keep it private?

- The local study team will know your name and have access to your information.
- We will do our best to make sure no one outside the study knows you are part of the study.

- We will take your name off information and study samples that we collect from you during the study. We will give your information and study samples a code, so that no one outside the study team can identify you.
- Information collected by the app will be automatically uploaded from your phone to our secure database. This upload will only occur when you are on a private wireless or cellular network. When you are not on a private wireless network (that is, a public network, like a coffee shop), then the information will be stored locally in the app until you are on a private wireless or cellular network, at which point it will be automatically uploaded to our secure database. Once the information is uploaded to our secure database, it will be deleted from the app.
- Information uploaded from the app will be stored on our secure database under your study ID. The database will not contain your name, phone number, email, or address. The only potentially identifying information collected in the database is your GPS data, discussed below.
- The smartphone information we collect will give us information about places you visit, which could be used to discover where you live or work. To prevent this, we will never share the "raw" smartphone GPS data. Instead we will recode the GPS data into a measure describing your area. For example, the Neighborhood Distress Scale summarizes neighborhood characteristics (crime rates, unemployment rates, etc.) as a three-point scale ("low, medium, high"). This will prevent people outside the study team from identifying exact locations from the smartphone GPS data.
- Some information you provide will be stored in the OptiMAT app. These include:
    - ✓ Your email (for technical support requests)
    - ✓ Your phone number (for you to call your emergency contacts)
    - ✓ Names, phone numbers, and relationships of your emergency contacts (for you to call in an emergency)
    - ✓ Your birth month and day (for an optional birthday message)
    - For your privacy, we will only store this information in the OptiMAT app on your phone and not in our database. You can change or delete this information at any time. Deleting the app from your phone will also delete this information.
- When we share the results of the study in publications and presentations, we will not include your name or anything else that could identify you.
- There are people who make sure the study is run the right way. These people may see information that identifies you. They are
    - ✓ National Institutes of Health (NIH)

  - ✓ OHRP (Office for Human Research Protections), a federal agency
  - ✓ UAMS Institutional Review Board
  - ✓ Other institutional oversight offices

- This study is supported by the National Institutes of Health. As a result, this study has a Certificate of Confidentiality. With this Certificate, the researchers cannot be forced to disclose information that may identify you, even by a court subpoena, in any federal, state, or local civil, criminal, administrative, legislative, or other proceedings. The researchers will use the Certificate to resist any demands for information that would identify you, except as follows:

  - ✓ The Certificate cannot be used to resist a demand for information from personnel of the United States Government that is used for auditing or evaluation of Federally funded projects or for information that must be disclosed in order to meet the requirements of the federal Food and Drug Administration (FDA).

  - ✓ You should understand that a Certificate of Confidentiality does not prevent you or a member of your family from voluntarily releasing information about your involvement in this research. If an insurer, employer, or other person obtains your written consent to receive research information, then the researchers may not use the Certificate to withhold that information.

  - ✓ The protection offered by the Confidentiality Certificate does not stop us from voluntarily reporting information about suspected or known sexual, physical, or other abuse of a child or older person, or a subject's threats of violence to self or others. If any member of the research team is given such information, he or she will make a report to the appropriate authorities.

Where and for how long will my information be kept?

- Your information will be labeled with an anonymous study code. Electronic information will be kept on our secure computer server. Paper information will be kept in your participant folder, stored behind two locked doors.
- Once we give your information a code, we will keep the key to this code in a locked filing cabinet in a room separate from the study information.
- Only research staff will be able to link your study code to you.
- Your coded information will be kept indefinitely. The study key linking your information to your identity will destroyed seven years after the closure of the study.
- We will not put information about you from the study in your medical record.

If I stop being in the study, what will happen to my information collected in the study?

- You can stop being in the study at any time. We will not collect any new information about you after you leave the study, but we will keep any information that you have already provided. When you delete the OptiMAT app, you will delete any information in the app which has not already been uploaded to our server. However, any information already uploaded to the server will not be deleted.

Will my information or samples from the study be used for anything else, including future research?

Yes. Any de-identified information (no names) we collect may be shared with other investigators not at UAMS. Dr. James may also share your information with large, public databases that allow scientists like Dr. James to share their data and methods. These databases store uploaded data indefinitely and do not allow individual participants to withdraw their data. However, all data will be de-identified prior to sharing. This means that all identifying information (such as names, dates, addresses, etc.) will be removed from data prior to sharing. We will share your coded data, but we will never share the study code linking your information to you.

Will you tell me the results of the study?

No. We will not tell you about your specific results, because that will require identifying individual subjects from our data. However, we plan to publish the results in an academic journal. (What we publish will not include anything that can identify you though.)

Will you tell me anything you learn that may affect my health?

Yes. If we learn something about you that might be important for your health, we will tell you.

What if new information comes up about the study?

We will tell you if we learn anything that may change your mind about being in the study.

What will happen at the end of the study?

We ask you to be in our study for 6 months. If you are in the app arm, you may continue to use the app after 6 months, until funding for this study ends (estimated fall 2026). If you were not selected for the app group, you may be invited to use the app after the 6 months, if the scientific team finds early evidence that the app may be helpful.

Where can I find more information about this clinical trial?

A description of this clinical trial will be available on http://www.ClinicalTrials.gov, as required by U.S. law. This website will not include information that can identify you. At most, the website will include a summary of the results. You can search this website any time.

Who has a financial or intellectual property interest in OptiMAT?

OptiMAT was developed by Dr. James and the study team. UAMS will copyright and trademark the OptiMAT intellectual property on behalf of Dr. James and the study team. UAMS and the study team do not have a current financial interest in OptiMAT, since OptiMAT is being evaluated in this study at no cost to you. However, UAMS, Dr. James, and the study team may have a future financial interest in OptiMAT, if OptiMAT is marketed for commercial use in future studies.

What if I have questions?

- Please call the head researcher of the study – Dr. Andrew James at (501) 420-2653 – if you
  - ✓ have any questions about this study
  - ✓ feel you have been injured in any way by being in this study
- You can also call the office at UAMS that supervises research if you cannot reach the study team, have questions about your rights as a research participant, or want to speak to someone not directly involved with this study. To do so, call the UAMS Institutional Review Board at 501-686-5667 during normal work hours.

By signing the document, I am saying:

- ✓ I agree to be in the study.
- ✓ I know that joining this study is voluntary.
- ✓ Someone has talked with me about the information in this form and answered all of my questions.

I know that:

- ✓ I can stop being in the study at any time and nothing bad will happen to me.
- ✓ I can still get medical care at UAMS and affiliated clinics no matter what I decide.

- ✓ I can call the office that supervises research (UAMS Institutional Review Board) at 501-686-5667 if I have any questions about the study or about my rights.
- ✓ I do not give up any of my legal rights by signing this form.

I agree to be part of this study:

______________________________ ______________________________
Your name (please print) Your signature

____
Date

______________________________ ______________________________
Printed name (person obtaining consent) Signature (person obtaining consent)

____
Date

I agree to be contacted for future research related to this study.

___ YES ___ NO

______________________________ ______________________________
Your name (please print) Your signature

____
Date

Study Title: Neurocognitive Mechanisms Underlying Smartphone-Assisted Prevention of Relapse in Opioid Use Disorder

Principal Investigator:
George Andrew James, PhD
University of Arkansas for Medical Sciences
4301 W. Markham Street, Slot # 554
Little Rock, AR 72205
Telephone: 501.526.8345
Email: GAJames@uams.edu

Sub-Investigator(s):
Ronald G. Thompson Jr., PhD
University of Arkansas for Medical Sciences
4301 W. Markham Street, Slot # 554
Little Rock, AR 72205
Telephone: 501.526.8100
Email: RGThompson@uams.edu

Mary J. Bollinger, PhD
University of Arkansas for Medical Sciences
4301 W. Markham Street, Slot # 554
Little Rock, AR 72205
Telephone: 501.526.8100
Email: MJBollinger@uams.edu

Michael Mancino, MD
University of Arkansas for Medical Sciences
4301 W. Markham Street, Slot # 554
Little Rock, AR 72205
Telephone: 501.526.8100
Email: MancinoMichaelJ@uams.edu

Clint Kilts, PhD
University of Arkansas for Medical Sciences
4301 W. Markham Street, Slot # 554
Little Rock, AR 72205
Telephone: 501.526.8100
Email: CDKilts@uams.edu

Keith Bush, PhD
University of Arkansas for Medical Sciences
4301 W. Markham Street, Slot # 554
Little Rock, AR 72205
Telephone: 501.526.8100
Email: KABush@uams.edu

Study location: University of Arkansas for Medical Sciences and affiliated MOUD clinics

Background and Rationale

The United States is experiencing an opioid crisis. Opioids were involved in 49,860 overdose deaths (over 135 people daily) in 2019, accounting for 71% of all US overdose deaths. Rural states are particularly impacted by this growing epidemic, as greater opioid prescription rates and less accessibility to healthcare resources have resulted in opioid-related mortality rates up to four times greater than urban areas. As a rural state, Arkansas is no exception, having the second highest opioid prescribing rate in the nation (75.8 opioid prescriptions per 100 residents as compared to national average of 43.3 in 2020).

The most efficacious therapy for opioid use disorder (OUD) is medication for OUD (MOUD) which uses opioid substitution (primarily methadone or buprenorphine) to alleviate craving and withdrawal symptoms to promote abstinence. Clinical trials have reported opioid abstinence rates as high as 75% during short-term inpatient MAT. But an estimated 60% of OUD patients relapse within one year of initiating outpatient MAT therapy. Adjunctive medication therapies during MAT (such as extended-release naltrexone) help attenuate relapse rates (reducing rates to ~40%), but these adjunctive therapies likewise suffer from limited availability in rural areas.

Telemedicine has emerged as a potential solution to the geographic barriers limiting healthcare access in rural areas. Specifically, smartphone applications ("apps") have been developed which administer brief motivational interventions to aid weight loss, smoking cessation, and alcohol use reduction. These app-based interventions combine self-monitoring of caloric intake/drug use with personalized feedback to shape users' behavior. However, this technology has not been applied to OUD in rural states, where rates of OUD are increased relative to population size and MOUD access is limited by geographic and financial barriers.

Therefore, we developed OptiMAT ("Optimizing Medication Assisted Treatment"), a novel app-based intervention to reduce opioid relapse during outpatient MOUD. OptiMAT provides: self-monitoring of daily opioid use, opioid craving, and mood; personalized feedback on goal attainment; charts depicting self-assessments over time; health information, including nearby abstinence-supporting resources; and daily reminders to complete logging activities. A small pilot trial (N=33) of an OptiMAT prototype among MOUD patients at the UAMS Center for Addiction Services and Treatment (CAST) found that ~20% of patients receiving MOUD alone lapsed in the first 3 months of treatment. Comparatively, 100% of CAST patients using OptiMAT remained opioid abstinent during the same period, suggesting strong potential for further study.

Overview of the OptiMAT app

The OptiMAT app has three primary functions: 1) daily tracking (self-monitoring) of opioid use, opioid craving, opioid withdrawal, and mood; 2) personalized feedback and encouragement, including charts depicting progress over the past week; and 3) location-based just-in-time intervention when participants enter personalized areas of high risk for relapse. OptiMAT offers additional features to promote abstinence, such as a) a Tip Bank with suggestions to avoid craving (e.g "When you feel cravings, try going for a walk or doing household chores to distract you from your cravings."); b) links to external resources (e.g. the NIDA website describing opioids and other drugs of abuse); c) tools to increase app and treatment engagement (e.g. alarm reminders to complete the daily tracking log or take their daily medication).

Specific Aims

Aim 1. Evaluate efficacy of OptiMAT for reducing illicit opioid lapse and relapse during outpatient MOUD. OptiMAT is an adaptation of HealthCall-S,[1] an app we showed in a previous RCT to reduce both alcohol misuse and unprotected sex among homeless young adults.[2] We will recruit 336 OUD patients initiating outpatient MOUD to participate in a two-arm RCT (1:1 randomization). One arm would be assigned to use the OptiMAT app in addition to outpatient MOUD while the second arm will receive outpatient MOUD only (i.e., Monitoring Only arm). <u>Hypothesis: Supported by pilot data, the percentage of clinically-acquired urinalysis tests positive for opioid misuse across 6 months of MOUD will be significantly less for the OptiMAT arm than the Monitoring Only arm</u>.

Aim 2. Identify neurocognitive mechanisms underlying app-based intervention outcomes. Despite the demonstrated efficacy of app-based interventions, we have a limited mechanistic understanding of how they work. To improve upon these interventions, we must first understand the mechanisms underlying individually varying treatment response. Functional MRI (fMRI) is a valuable tool for mapping neural processing changes that encode treatment outcomes, such as patterns of functional brain organization which predict subsequent response to interventions in depression.[3] 120 participants (60 from each arm, OptiMAT and Monitoring Only) will undergo a longitudinal fMRI substudy identifying neural networks subserving two relapse-related cognitions (attentional bias to drug craving cues, and processing of opioid and monetary rewards) at baseline, 2 months and 4 months post-enrollment. <u>Hypothesis: longitudinal changes in the brain-behavior relationships encoding these cognitions will independently predict individual differences in OptiMAT-related outcome.</u>

Exploratory Aim 3. Evaluate use of OptiMAT-GEMA to reduce opioid relapse. Drug craving has a strong contextual dependence.[4–6] At enrollment, OptiMAT participants will report three locations ("hot spots") where risk of opioid relapse is greatest (e.g. where they buy opioids). OptiMAT will use location-based services to identify when participants enter and remain within these personalized hot spots and send an intervening push notification (consisting of an opioid craving self-assessment, followed by an intervention such as phoning the Arkansas Opioid Crisis Hotline, as needed). Post-hoc analyses will evaluate the qualitative and quantitative factors (i.e., did user respond to notification, duration spent in hot spot) that determine individual differences in whether GEMA boosts OptiMAT-related relapse prevention. <u>Hypothesis: OptiMAT participants who respond to GEMA "just-in-time" intervention will have fewer percent positive urinalysis results than participants who do not respond to the intervention.</u>

Study Design and Procedures

<u>Important Note</u>: UAMS research staff will conduct all study activities (recruitment, interviews, app training, MRI data collection, follow-ups), including activities conducted at UAMS-affiliated clinics.

<u>RCT study design</u>: We will recruit 255 patients with opioid use disorder who have initiated outpatient MOUD to participate in a two-arm randomized control trial (RCT). Participants will be randomized 1:1 to either the MAT only arm (treatment as usual) or MAT + OptiMAT arm (active intervention). A separate randomization scheme will be developed for each study site, to ensure balanced recruitment into the study arms across all sites. Participants outcomes will be followed with monthly Time-Line FollowBack phone assessments of opioid misuse for 6 months. Please see **Ethical Considerations** for information about RCT Recruitment, Randomization, and Retention.

<u>MRI sub-study design</u>: A subset of participants in the RCT (N=120, 60 per arm) will be invited on a first-come first-serve basis to a longitudinal MRI study, measuring brain changes with treatment at baseline, 2 months, and 4 months later. The goal of the MRI sub-study is to understand how the brain changes with recovery, and how the smartphone may facilitate those brain changes. Please see **Ethical Considerations** for information about MRI sub-study Recruitment, Randomization, and Retention.

Participants in the MRI sub-study will attend an MRI Intake visit (~3 hours) and 3 longitudinal MRI visits (at Baseline, 2 months, and 4 months; ~2 hours each). Each MRI visit will be conducted at the Brain Imaging Research Center (BIRC). Participants will undergo structural MRI imaging of brain anatomy at each MRI visit. Participants will also undergo MRI imaging of brain function during the following tasks:

Resting state: We will study "baseline" brain activity as participants passively view a white fixation cross upon a blank screen. This task will be conducted across two scans of total duration ~15 min.

Counting Stroop task: The Counting Stroop task[7] measures the amount of distraction (known as "attentional bias") for drug-related stimuli and incongruent cognitive stimuli. At MRI Intake, participants will provide 6-12 personalized cue words associated with their opioid use behaviors that trigger their opioid

craving. The research coordinator will work with participants to rank and select the six words that induce the greatest opioid craving. These words may include opioid-related paraphernalia, first names or nicknames of their dealers or friends they used opioids with, places where they frequently used opioids, or sensations associated with using opioids. During the task, participants will view 1–4 identical words presented in a vertical column and indicate, as quickly as possible, the number of words shown by pressing the corresponding button (index, middle, ring, or pinky fingers for 1–4, respectively). 288 trials (50%) will consist of neutral words (e.g., desk, phone). 144 trials (25%) will be Incongruent Stroop (iStroop) stimuli, consisting of the words "one" through "four" presented an incongruent number of times (e.g. the word "two" presented four times). 144 trials (25%) will be the personalized opioid use cues (oStroop) described above. Words will be matched for lexical properties such as length and frequency of use. Trials (2.3 sec) will be presented in a pseudorandom fast event-related design optimized to model the BOLD response for each trial type with the program Optseq.[8] The task will consist of 2 runs of ~8 min each, including 3 fixation blocks (25s) per run as a simple baseline control.

Opioid cue reward imagery task: Participants will view images from the Methamphetamine and Opioid Cue Database (MOCD),[9] a recently curated dataset of methamphetamine, opioid, and non-drug cues with normative ratings of valence, arousal, and craving. Participants will passively view images of opioids and non-drug cues (no methamphetamine cues) as a fast event-related paradigm. Each stimulus will be presented for 2 s, followed by a 2-6 s inter-trial interval (ITI) consisting of a fixation cross. Approximately 80 stimuli will be presented. The task will consist of two runs of ~5 min each. At the end of each MRI session, participants will rate each stimulus for valence, arousal, and craving intensity on a 7-point Likert scale.

Monetary reward task: Participants will undergo the Monetary Incentive Delay Task (MIDT),[10] a standardized task for assessing neural activity during reward anticipation, reward receipt, loss anticipation, and loss receipt. Each trial consists of a cue indicating whether they may win money (pink circle, showing $0.20 or $5), not lose money (yellow circle, showing $0.20 or $5) or neither win nor lose (blue triangle showing $0). After a variable delay (1.5-4s), the shape turns black for ~300ms before disappearing. If the participant presses the button before it disappears, they win (or don't lose) the displayed amount. If they are too slow, then they do not win (or lose) the displayed amount. The ~300ms delay is titrated by participant performance so that they will win ~60% of trials. Participants are anticipated to win ~$10 per run, or ~$20 total. They will win at least $1 per run to encourage participation. The task will consist of two runs of ~5 min each.

Study Population

336 participants ages 18+ enrolled in MOUD treatment will be recruited for this RCT. Please see <u>Ethical Considerations, Recruitment</u> for a detailed description of how research staff will approach and consent patients to be participants in this study.

<u>Inclusion Criteria</u>

- Male or female
- Age 18+ (for RCT) or 18-50 (for MRI substudy)
- Enrolled in outpatient MOUD treatment at UAMS CAST or a MOUD clinic affiliated with the UAMS MATRIARC program
- Currently in Phase I of MOUD treatment, defined as having weekly on-site Suboxone administration with a 1-week supply of daily take-home medication
- Has completed intake visit and at least one weekly individual therapy session

<u>Exclusion Criteria</u>

- (MRI substudy) Medical history: A history of neurological, cardiovascular, or infectious disease would exclude study participation. A loss of consciousness of 20 or more min or other evidence of brain trauma also would be exclusionary.

- (MRI substudy) Pregnancy: A positive test for pregnancy prior to fMRI would exclude participation.
- (MRI substudy) MRI contraindications: Exclusion criteria for MRI include (1) the presence of non-removable internal (e.g., cardiac pacemakers, aneurysm clips, artificial joints) or external (e.g., piercings, orthodontics) ferromagnetic objects; (2) claustrophobia in a confined MRI environment; (3) medications that interfere with hemodynamic coupling (e.g., beta blockers); (4) hypersensitivity to loud noise; or (5) morbid obesity, including exceeding the MRI bed's weight limit of 500 lbs.

Risks and Benefits

Risks Related to Study Participation: Sources of risk stem from the collection and maintenance of material sources. NOTE: These risks apply to both in-person assessment and assessment via the OptiMAT app.

<u>Risks associated with loss of confidentiality</u>. Potential risks to subjects related to loss of confidentiality include social, employment, and legal consequences following self-report of their drug use, childhood adversity, and other personal histories.

<u>Risks associated with assessment of mental health and traumatic event exposure</u>. One potential psychological risk of the proposed study is the possibility that some subjects might experience distress or become offended when asked questions pertaining to stressful situations, victimization and mental health history. Many people assume that asking such questions produces substantial distress, particularly in research settings. However, the empirical literature suggests that this risk is minimal, and that individuals with traumatic event histories actually report obtaining positive benefits from their participation in studies where traumatic event history is assessed. Nevertheless, there is a specific protocol, should a subject become distressed as a result of participation in this study. This is discussed in detail below.

<u>Risks associated with assessment of drug craving or cue-induced drug craving</u>. Similarly, subjects might experience distress when asked to report their current level of drug craving, or when inducing craving through the Stress Imagery fMRI task. Although this has been a rare occurrence in past studies (<5% of participants), there is a specific protocol in place to manage subject's study-related craving. This is discussed in detail below.

<u>Risks associated with assessment of suicidal ideation</u>. During the clinical interview, questions regarding suicidal ideation are asked. This may elicit distress from subjects; however, there is a specific protocol should a subject report suicidal ideation, intent, or plan, and a protocol to decrease distress, described below.

<u>Risks associated with MRI acquisition</u>. Potential risks to subjects related to MRI include the motion of internal ferromagnetic objects, the potential of external ferromagnetic objects to serve as projectiles, claustrophobic experiences, and possible anxiety. The safety of 3T MRI to unborn offspring is unknown.

<u>Risks associated with GPS-driven location services</u>. Potential risks to subjects include disclosing locations for opioid purchase or use. Additionally, the "just in time" notification may be annoying or distracting as participants are passing through the area.

Data Safety Monitoring Board (DSMB)

We will establish a DSMB to monitor treatment outcomes. The DSMB will consist of:

- Allen Sherman, Ph.D. Clinical Psychology: Chair of the UAMS IRB
- Paula Roberson, Ph.D. Biomathematics: Chair of the UAMS Department of Biostatistics
- Xiawei Ou, Ph.D. Physics (MRI): Associate Professor in UAMS Department of Radiology

The DSMB will meet annually to review study-related AEs and SAEs. The PI will conduct interim Aim 1 analyses (comparing rates of opioid use between OptiMAT app vs. Monitoring only arms) and present these findings to the DSMB. The DSMB will make recommendations concerning study stoppage, as warranted by

data and ethical considerations. For example, if the OptiMAT app shows clear superiority over Monitoring, then the Monitoring arm may be closed (with continued enrollment into the OptiMAT arm) to allow greater power for understanding the app's mechanisms of function. Conversely, if the OptiMAT intervention shows no difference (or even worse outcome), the DSMB may recommend suspending enrollment to the OptiMAT arms but with continued monitoring to understand why the intervention failed and warrant future improvements in app-based intervention. DSMB recommendations and PI's response will be shared with NIH via annual progress reports.

Data Handling and Recordkeeping

The Principal Investigator will carefully monitor study procedures to protect the safety of research subjects, the quality of the data and the integrity of the study.

Protection Against Loss of Confidentiality.

OptiMAT assessments: The OptiMAT app will serve as a portal to a secure database storing all data entered by the participants. Any data entered into the OptiMAT app will be uploaded by secure HTTPS connection to a HIPAA-compliant, HITRUST certified secure database managed by Enqbator. If the smartphone is on a secure private wireless network (like their home network or their provider's cellular network), then the entered data will automatically upload to the server. If the smartphone is not on a secure private wireless network (e.g. a public network, like at a coffee shop), then the data will be stored on the phone locally until the smartphone is connected to a private wireless network or cellular network, at which point it will be automatically uploaded to server. This transfer process will happen automatically. Second, the participant will always be able to delete the OptiMAT app to prevent loss of confidentiality without losing data collected on the server. Third, study staff will require participants to enable passcode protected lockscreens on their phones, and warn them about potential loss of privacy if they disable the passcode protected lockscreens. Finally, OptiMAT will store data using only study codes (no PHI) to further protect privacy and confidentiality.

Enqbator server and data access: Enqbator retains no rights to the study data uploaded to its server. At the conclusion of this study, the PI will download all study data from the Enqbator's server, and Enqbator will delete the data on the server. All data on Enqbator server will be stored using study ID. The only potentially identifying information on the server is GPS location, which may be used to infer a home address or work address. Enqbator and UAMS have entered into a Master Services legal agreement specifying that Enqbator will "keep the Disclosing Party's (UAMS) Confidential Information confidential, and will not use or disclose such information to any third party for any purpose except (i) as expressly authorized by the Disclosing Party in writing, or (ii) as needed to fulfill the Receiving Party's obligations under this Agreement."

In-person assessments: To protect the identity of research volunteers, identifying contact information would be stored in a file (electronic) or in a locked cabinet (paper consent forms) separate from experimental data forms. All experimental data would be de-identified using an alphanumeric study code rather than a name or other identifying information. All data collection forms would be stored in a locked cabinet in the in a key card-protected room in the BIRC. Computer data records would be stored in password-protected network drives accessible only by study personnel. Data would be stripped of all identifiers, including the stripping of facial features from the anatomical MRI data. As a further measure to protect against the compelled disclosure of personally identifiable information, the investigative team has a NIDA-supported Certificate of Confidentiality. All personnel involved in the conduct of the proposed research would comply with the applicable Federal regulation for the protection of human subjects or, if no such federal regulation is otherwise applicable, they would comply with 45 CFR Part 46.

UAMS IRB policy considers all study personnel as mandated reporters, requiring that study personnel report incidents of child abuse and neglect to the Arkansas Department of Human Services. Study personnel must also report intentions to hurt others to local authorities. Subjects will be informed of this during the consent

process, and that incidents of child abuse/neglect or intentions to harm others are not covered by the Certificate of Confidentiality.

Assessment of Opiate Craving and Cue-Induced Craving.

OptiMAT craving assessments: The opiate craving Likert scales used by OptiMAT are accompanied by personalized feedback. Participants rating high craving intensity will be prompted with personalized feedback, including tips to reduce craving. OptiMAT will also provide resources such as the Arkansas 2-1-1 Crisis hotline.

Cue-induced craving: The Stress Induction fMRI task includes an in-scanner relaxation script to calm participants after re-experiencing their stressful life events. Research staff are trained in clinical interviewing techniques for identifying and easing distress. At the conclusion of MRI scanning, participants whom report high craving or high-risk of relapse will be referred to Dr. Mancino and the UAMS PRI Center for Addiction Services and Treatment for arranging appropriate addiction-based therapy.

Assessment of Traumatic Event Exposure: There is a very low probability that any subject will experience more than minimal distress during the interviews. Research staff are trained in clinical interviewing techniques for identifying and easing distress. If while ascertaining a subject's current condition the need for clinical intervention is determined by staff, the participant will be referred to Dr. Mancino and the UAMS PRI Center for Addiction Services and Treatment for arranging appropriate trauma-focused therapy.

Procedures for Suicidal Subjects: Individuals who are actively suicidal, defined as intending to hurt or kill themselves in the acute time frame, will receive immediate crisis counseling services by referral to the UAMS Emergency Room and the UAMS psychiatrist on call there for such emergencies. The treatment response may include hospitalization. These subjects will be ineligible for the experimental session.

Protection Against Risks Associated with MRI: Pregnancy testing would be administered to all subjects prior to MRI with a positive test result representing an exclusion criterion to avoid potential fetal health risks. Any history of claustrophobia would be assessed with subjects acclimated to the scanner environment using a MR simulator to assess and minimize anxiety. The BIRC MRI screening instrument would be used to screen for any internal or external ferromagnetic objects. If not removable, such objects would represent a basis for study exclusion. A handheld ferromagnetic detector (SafeScan®) would be used over each subject prior to MRI scanning to provide a safeguard against unreported internal or external ferromagnetic objects. It is the policy of UAMS Brain Imaging Research Center performance site that all subjects would be informed prior to consent that the MRI acquisitions are for research rather than clinical purposes and that unintended MRI findings (e.g., AV malformations, tumors) would not be assessed and therefore not reported. These disclaimers are explicitly stated in the IRB-approved consent form for the project. In the rare event that such a finding is noted the images in question would be referred to a clinical neuroradiologist and the subject contacted for follow-up if he/she feels it is clinically prudent.

Upon study closure, the data will be de-identified, curated using Open Science frameworks including the universal BIDS data format, and made a publicly available data repository via DataLad. The study key linking participant identity to study ID will be retained for seven years after publication of the RCT results per UAMS IRB policy, after which it will then be destroyed.

Protection Against Risks Associated with GPS-driven location services. Several approaches will be used to minimize risks associated with use of GPS location services to deliver "just-in-time" interventions in Aim 3. First, areas of high contextual risk will be defined using generic intersections rather than specific street addresses, so as to better anonymize where illicit opioid sales or misuse is taking place. Second, the just-in-time intervention will only occur when participants linger in the area for greater than 5 minutes, to prevent false alarms when participants are just passing through the area. Third, this aim will rely upon geofencing, which is managed on Enqbator's secure server and not within OptiMAT. The server will quietly "ping" each phone once per minute for its GPS location, determine when and for how long a phone stays within a personalized geofenced location, then trigger the just-in-time intervention accordingly. This means that the hotspot of high

risk of relapse is not stored within the participants' OptiMAT app, and thus cannot be displayed or accessed by individuals using the app, only research staff. This also means that closing OptiMAT won't turn off the just-in-time intervention. Finally, participants will always have the option of disabling GPS by entering airplane mode or simply turning off their phones. Enqbator's geofencing software will detect and record when phones are inaccessible due to disabling GPS services, which will later be compared against the monthly Time-Line FollowBack phone calls to determine if opioid misused occurred during one of these GPS disabled moments.

An additional concern about GPS-driven location services relates to de-identifying GPS data prior to data sharing. Third-parties could infer identifying information (including home address) from the precise GPS data. To counter this, all GPS data will be de-identified prior to data-sharing. The "best practice" for de-identifying GPS data is evolving, and has ranged from scrambling latitude and longitude (which is not suitable for this work, since all participants are in one area), recoding all variables to distance from a fixed point (which is also not suitable, since locations could be triangulated from their UAMS appointments), or restricting data to census block (which we believe is too specific). Prior to sharing data, we will de-identify all GPS data by recoding it into two indices: the Neighborhood Distress Index (a three-level scale, "low, medium, high") and a binary variable indicating if subject was inside or outside one of their personalized hotspots. Since the best practice for de-identifying GPS data is constantly evolving, **we will consult further with the IRB at project closure prior to sharing any location-based GPS data.**

Data Analysis

Power analysis for sample size determination. As brief interventions, smartphone apps have large effect sizes (Cohen's δ) for aiding weight loss ($\delta \geq 0.60$), but weaker effects for aiding smoking cessation ($\delta \approx 0.16$–0.28) and alcohol reduction ($\delta \approx 0.23$-0.40). Our pilot OnTrack study suggested similar effect sizes for reducing alcohol and marijuana use (both $\delta \approx 0.26$) and greater effect sizes for reducing risky sexual behavior ($\delta \approx 0.70$).[2] A clinical trial using the CHESS-A smartphone app[11] estimates an effect size for reducing opioid use of $\delta = 0.35$ – an effect size comparable to reducing use of other drugs of abuse. Assuming Cohen's $\delta = 0.35$ and 1:1 randomization into the OptiMAT vs Monitoring arms, $\alpha = 0.05$ and $\beta = 0.80$ could be attained by accruing N=204 participants. Assuming a 20% drop-out rate and stratification of random study arm assignment across 12 variable levels (up to 3 recruitment sites x 2 chronic pain levels x 2 levels of comorbid use of marijuana or alcohol), this accrual goal could be reached with enrollment of N=336 participants.

Functional neuroimaging approaches to defining brain-behavior relationships have a relatively high effect size: our previous work[7] modeling the neural processing correlates of attentional bias for cocaine-related cues reported correlation rho=0.359 between brain activity and behavior, corresponding to Cohen's $\delta = 0.77$. We reported a more conservative behavioral effect size ($\delta = 0.57$) for within-subject slowing of RT in response to cocaine cues.[12] Assuming that participants who benefit from OptiMAT show a normalized AB effect (i.e. ≈70ms reduction in opioid cue AB at 6 months post-enrollment), then $\delta = 0.57$, $\alpha = 0.05$ and $\beta = 0.80$ could be attained by recruiting N=26 treatment responders per arm. Since we won't know who will/won't respond at intake, and given that approximately 40% of opioid patients undergoing outpatient MAT will relapse within 6 months, we would need to recruit N= 26/(1.0-0.4) = 44 per arm to test if successful treatment response corresponds to a reduction in the behavioral and neural correlates of opioid-related attentional bias. Assuming an overall 20% loss-to-follow-up rate, we will recruit N=60 participants for each arm of the Aim 2 longitudinal fMRI substudy.

Table 3. Study-investigated variables.

Domain/Variable	Measure/Description	Timepoints
**Primary and Secondary Outcome variables**		
	**Urinalysis Tests**: Acquired clinically by CAST	Weekly

Opiate Use (and Other Drug Use)	**Time-Line Follow-Back (TLFB)** for Opiates, Alcohol, Marijuana, and Other Drugs: Number of drug-use days per month, episodes/amounts of drug use.	Intake; Monthly Phone Call
	**OptiMAT Daily Ratings**: Number of drug-use days per month	Daily (OptiMAT only)
Addiction Severity	**Structured Clinical Interview for DSM-5 (SCID-5)***: screens for non-exclusionary comorbid psychopathology and assessed severity of opioid use disorder and other drug disorders	MRI Intake visit
Craving Severity	**OptiMAT Daily Ratings**: measures daily drug craving severity	Daily (OptiMAT only)
Withdrawal Severity	**OptiMAT Daily Ratings**: measures daily withdrawal symptom severity	Daily (OptiMAT only)
Geographical Location	**OptiMAT Location-based Services**: records entries into personalized high lapse-risk areas and total duration spent in areas	Every Minute (OptiMAT only)
**Variables Mediating or Moderating Outcome**		
Cellphone Use	**Percent days of OptiMAT usage**	n/a
Demographic	**BIRC Demographic form**: age, sex, race/ethnicity, education, income to need	Intake
Attentional Control	**D-KEFS Color-Word (Stroop) Test***: measures selective attention	MRI Intake visit
	**fMRI Counting Stroop task**: neurocognitive measure of attentional control vs. incongruent and personalized drug-cue distractors.	All MRI visits
Cue-Induced Craving	**Brief Substance Craving Scale**: assess current craving intensity for opiates	All MRI visits
	**fMRI Craving task**: neurophysiological measure of response to normative images of opioid use	All MRI visits
Reward Processing	**fMRI Money task**: neurophysiological measure of anticipation and receipt of rewards and losses	All MRI visits
Impulsivity	**Barratt Impulsiveness Scale (BIS-11)**: measures domains of impulsivity	MRI Intake visit
Verbal Intelligence	**Receptive One-Word Picture Vocabulary Test (4th ed)***: receptive vocabulary	MRI Intake visit
Working Memory	**WAIS-IV Digit Span test***: measures verbal working memory	MRI Intake visit
**Control Variables and/or Potential Confounding Variables**		
Early Life Adversity	**Childhood Trauma Questionnaire**: assesses childhood abuse and neglect	MRI Intake visit
Resilience	**Connor-Davidson Resilience Scale**: measures resiliency from childhood trauma	All MRI visits
Attention	**Continuous Performance Test (CPT)***: measures attention capacity	MRI Intake visit
Pain Comorbidity	**PROMIS Pain Interference Short form v1.0 4a**: assesses pain interference	Intake, Monthly Phone Call
Socio-economic status	**Sociodemographic Questionnaire**: assesses perceived SES	Intake, 6 months
***These assessments are proprietary and cannot be uploaded to CLARA.**		

At this time, there is insufficient effect size and variance data to conduct an informed power analysis for Exploratory Aim 3, using GEMA to support location-based intervention. We will report post-hoc effect sizes from data acquired for this Aim.

Aim 1. Evaluate efficacy of OptiMAT for reducing opioid misuse during outpatient MAT.

Rationale, hypothesis, and expected outcomes. Our primary hypothesis is that the complementary lapse prevention features of app-delivered OptiMAT will boost the opioid abstinence promoting effects of MAT for men and women with OUD. The expected experimental outcome associated with confirmation of this hypothesis is that MAT-engaged participants in the OptiMAT arm will have significantly reduced opioid misuse (i.e. use of non-prescribed opioids) compared to participants in the Monitoring arm. Xiaotong Han MS, a staff statistician in the department of Psychiatry, will conduct these analyses comparing RCT arms. To ensure study integrity, Mrs. Han will be blind to group membership and is not directly or indirectly supervised by the PI or other faculty on this study.

Data analysis plan. Primary outcome variable is **percent of weekly urinalysis tests that are negative for non-prescribed opioids** during six-months of MAT treatment. These urinalysis results will be acquired at clinical visits and abstracted from participants' medical records. Urinalysis drug tests will be acquired at each clinical visit, which occur weekly when participants initiate treatment but may occur less frequently (e.g. every two weeks) as participants maintain sobriety. We will calculate percent negative urinalysis tests out of total possible urinalysis tests to generate a Treatment Effectiveness Score (TES) describing participant outcome relative to other participants in the RCT. Note that TES treats missed urinalysis tests and positive urinalysis results equivalently, thus accounting for treatment cessation. TES also accounts for the frequency of urinalysis

tests, which varies as patients matriculate from Phase I (weekly) to Phases II-IV (biweekly to monthly) as patients demonstrate continued abstinence from opioid misuse.

General linear or generalized linear models (GLMs) will be used to examine associations between study arms and primary outcome (percent positive urinalysis tests) at the 6-month endpoint depending on whether the outcome is normally distributed or not. The model will include the indicator variable for study arm (MAT+OptiMAT vs. MAT-only) and the three stratification variables used for randomization (Site, Chronic Pain, and comorbid Alcohol or Marijuana Use) only first. Then the model will be run again with any additional covariates that were identified as being unbalanced between the study arms, and results will be compared.

Secondary analyses. Secondary analyses will include Kaplan-Meier survival analyses to assess differences between arms in (1) time to first lapse in opioid misuse, (2) time to relapse (characterized as having to return to a more intensive MAT phase due to multiple lapses), and (3) discontinuation of MAT for any reason (including voluntary drop-out, dismissal by CAST due to repeated treatment noncompliance, or loss-to-follow-up). We hypothesize that OptiMAT MAT participants will have longer times to lapse or relapse and lower rates of MAT discontinuation compared to Monitoring arm participants.

We will also conduct mediation analyses[13] to evaluate if demographic[14] (age, sex, education, SES, rurality), cognitive[15] (attentional control, working memory, verbal intelligence; acquired for MRI participants), or app-based variables[16] (usage frequency, craving self-ratings, withdrawal symptom severity) impact OptiMAT/MAT treatment outcome. Specifically, we will use the Kenny, Kashy, and Bolger adaptation of mediation analysis[17] testing (1) if mediating variable M is significantly correlated with predictor variable X (arm assignment), and (2) if M is significantly correlated with outcome variable Y (percent positive urinalyses). We will use the machine learning approach LASSO (Least Absolute Shrinkage and Selection Operator)[18,19] as a collinearity-resilient alternative to stepwise regression to identify the subset of demographic, cognitive, or app-related variables correlated with both X and Y, then independently test each LASSO-identified variable as mediator M for the relationship X→Y. Kenny, Kashy and Bolger's adaptation is chosen over Baron and Kenny's original postulation[17] because we choose not to assume that X→Y will be significant in absence of a mediator; i.e. the point biserial correlation between arm assignment (X) and percent positive urinalyses (Y) may initially be non-significant but become significant after including a mediating variable M (e.g. percent-days of OptiMAT use).

Potential problems and alternative approaches. The primary outcome variable is total percent positive urinalysis tests which will not capture temporal variation of response. We will therefore also collect Time-Line Follow-Back calendars (all participants) and daily self-reports (OptiMAT arm) of opioid use, which may offer greater temporal sensitivity (days-use per month compared to weeks-use per month). Although broad support exists for TLFB's reliability,[20–24] we will conduct Cronbach's α internal reliability analyses of TLFB, OptiMAT daily self-ratings, and clinic urinalysis drug screen results. In event of high consistency (Cronbach's $\alpha \geq 0.70$), TLFB reports will be used as a secondary outcome measure.

Aim 2. Identify neurocognitive mechanisms underlying OptiMAT intervention outcomes.

Rationale, hypothesis, and expected outcomes. Treatment-related reduction in attentional bias for drug cues predicts positive treatment response (i.e. reduced drug use),[25,26] as does reduction in drug craving.[27–29] We hypothesize that individual variation in how OptiMAT engages the neurobiological networks underlying these cognitions (attentional control, craving) will predict treatment outcomes. For both treatment arms, we predict that longitudinal changes in neurocognitive networks will correlate with positive treatment response as follows: 1) decreased attentional bias RT and frontocingulinsular network recruitment for opioid use cues (i.e. a "strengthened" network would confer a diminished conditioned incentive motivation for opioid misuse) and (2) decreased craving responses and ventrostriatal network recruitment for opioid use cue-related mental imagery. We will compare the two networks' ability to independently and collectively predict outcome, as well as the temporal precedence of these changes (i.e. if changes in one network at 2- or 4- months midpoint predicts change in the other network at 6-month endpoint). Our repeated-measures linear mixed effect model below will

also assess the moderating influence of study arm (OptiMAT, Monitor only) on brain–behavior relationships, thus inferring OptiMAT's mechanism of action.

Data analysis plan. As described above, ICA will dimensionally reduce task-related brain activity to 30-40 spatiotemporally distinct functional networks. For the event-related Counting Stroop task, linear mixed effect modeling will relate frontocinguloinsular network function to task behavior using AFNI's 3dLMEr via the equation (in Wilkinson notation):

BRAIN (dACC network) ~ Cue * RT * Session * TX_Resp * Arm * Sex + (Cue * RT * Session | Subj)

where Cue= cue condition (neutral, opioid, incongruent), RT= trial reaction time, Session=MRI session (intake, 3-month, or 6-month), TX_Resp= treatment response (percent positive opioid urinanalysis results at study endpoint), Arm = treatment arm (OptiMAT or Monitoring Only), and "(*X* | Subj)" indicates modeling independent regressors for each subject (i.e. treating subject as a random effect). We will fully expand this 6-way interaction. RT will not be mean centered because we expect RT to significantly differ between Cue conditions.

Expected results: Our pilot data (N=7) shows a Cue*RT interaction, with an attentional bias effect for both Opioid cues (mean= 36ms) and Incongruent cues (mean= 86ms) relative to Neutral cues – comparable to our findings in cocaine users.[7] We anticipate a 4-way interaction of Cue*RT*Session*TX_Resp, with the Cue*RT AB effect reducing from intake to later sessions (2-month, 4-month) as a function of treatment outcome. We include Arm to test if this reduction is more pronounced in OptiMAT arm than Monitoring only, thus identifying a putative mechanism for OptiMAT's clinical outcome. Sex differences have been reported for affective AB paradigms[30–32] but not drug cue AB; we will nonetheless include Sex as a factor to evaluate sex differences.

For the block-design Cue-Induced Craving task, 3dLMEr will model ventrostriatal (VS) network activity as:

BRAIN (VS) ~ Condition * Craving rating * Session * TX_Resp * Arm * Sex + (Cond * Craving * Session | Subj)

where Condition= imagery script (opioid, neutral), Craving rating is the post-script Likert rating of craving intensity, and all other variables are the same as the Counting Stroop task. Given literature support for sex differences in imagery-induced stress craving,[4–6,33,34] we anticipate Sex to be a significant factor. Furthermore, we will compare the Arm*Session interaction effect between the two LMEs to evaluate the temporal dynamics of these two networks with treatment.

Secondary analyses. The inclusion of treatment arm in task-based LMEs will assess how OptiMAT changes brain function and behavior relative to MAT Monitoring only, but does not address *how* OptiMAT induces those changes. To directly relate these neurocognitive mechanisms to the OptiMAT app, we will conduct mediation analyses to assess the specific roles of app features (e.g. overall usage, self-ratings, Tip Bank) that may mediate the predictive influence of change in opioid use (X) to brain activity and/or behavior (Y).

Potential problems and alternative approaches. The potential interdependence of mechanistic Aim 2 on a positive clinical response to OptiMAT use in Aim 1 is somewhat mitigated by the positive pilot study findings.

Comorbidities (including chronic pain) could be confounds to the proposed neuroimaging analyses. We seek to circumvent this confound by incorporating the PROMIS Pain Interference Scale and comorbid marijuana or alcohol use into our randomization plan. In addition, we have recently published methodologies for modelling the independent and comorbid influences of cocaine dependence and other psychiatric illnesses on resting-state subgenual cingulate connectivity,[35] and extended these findings to model comorbidity-related perturbations of the motor impulsivity network during the Stop Signal fMRI task.[36] We will conduct secondary analyses of comorbidity influences on neural representations/predictors of treatment response.

The complexity of neuroimaging analyses poses potential problems. First, we assume that brain-behavior relationships are constant throughout the recovery process. However, the anxiety disorder literature suggests that treatment-related reductions in attentional bias correlate not with reduced frontocinguloinsular activity but instead with compensatory recruitment of lateral prefrontal cortex for increased cognitive control.[37] To address this issue, we will extend our LME analyses to all 30-40 ICA components, with post-hoc FDR correction, to test if any other networks' recruitment varies with Session*Tx_Resp, indicating other adaptive treatment responses.

Finally, the addition of the 3 Month fMRI session permits longitudinal data-driven approaches such as latent growth curve modeling (LGCM) which classifies participant subgroups based on longitudinal urinalysis outcomes (e.g. continuous abstinence, abstinence then relapse, relapsers who achieve abstinence) and identifies neurocognitive predictors at Intake, 2-Months, and/or 4-Months that distinguish subgroups. We will employ data-driven LGCM in addition to the preplanned analyses above.

Exploratory Aim 3. Evaluate use of geographic ecological momentary assessment (GEMA) to further boost the effect of OptiMAT on MAT outcomes.

Rationale, hypothesis, and expected outcomes. Our primary hypothesis is that GEMA-driven intervention when lapse risk is high will further enhance OptiMAT's effects on MAT-related reductions in opioid misuse. The expected experimental outcome is that GEMA-driven intervention will reduce opioid misuse through location-prompted just-in-time intervention. As described above, participants remaining within a self-identified high-risk area for opioid use lapses for longer than 5 minutes will receive a text notification to rate their current opioid craving. OptiMAT would send personalized feedback based on the response. A response ≥ 7 (on a 10-point Likert scale) would be followed by a push notification allowing the participant to automatically dial Arkansas 2-1-1 and speak to a counselor to cognitively process the current craving episode and/or seek immediate referral to emergency services. As a comparison condition, we will also conduct random EMA of participant craving 2-3 times a week. As with GEMA, an EMA craving rating ≥ 7 would be followed with push notification to contact Arkansas 2-1-1, though unlike GEMA this notification would not be initiated by one's lapse risk location.

Data analyses. Primary outcome variable will be **self-reported opioid use** (binary, "yes/no") during a 24-hour period following the hotspot-triggered GEMA intervention. We will compare acute opioid use lapse rates after location-based GEMA intervention to acute lapse rates following random EMA craving assessments. We will evaluate qualitative and quantitative metadata surrounding GEMA intervention as predictors of acute opioid use. Logistic regression will assess if binary qualitative variables (such as "did participant rate craving?" or "if participant received an Arkansas 2-1-1 push notification, did he or she call?") or quantitative variables (such as craving rating or duration spent in the hotspot) predict significantly greater risk of acute opioid relapse.

Secondary analyses. The EMA control condition will enable us to control for location-based meta-data, such as zip-code based crime rates or rurality, to determine if acute opioid lapse is specifically driven by contextualized associations with the hotspot or could be attributable to other objective, geographical features of that location.

Potential problems and alternative approaches. A host of environmental variables not captured by the OptiMAT app (such as stressful life events) may also predict opioid misuse. The research coordinator will receive daily notifications of self-reported opioid relapse and will contact participants by phone within two business days to determine what additional qualitative factors may have contributed to the relapse. These exploratory variables could inform further development of the GEMA-based intervention; for example, participants' reports of entering hotspots with the intention of finding opiates in response to an earlier stressful situation would support the development of an earlier GEMA intervention (as participant approaches the hotspot) or more direct intervention (calling Arkansas 2-1-1 on participant's behalf automatically, in absence of push notification).

Ethical Considerations

This study will be conducted in accordance with all applicable government regulations and University of Arkansas for Medical Sciences research policies and procedures. This protocol and any amendments will be submitted and approved by the IRB as required.

Recruitment. Participants would be recruited from the UAMS Psychiatric Research Institute (PRI) Center for Addiction Services and Treatment (CAST) or a MOUD clinic affiliated with the UAMS MATRIARC program. Consistent with past and current IRB-approved recruitment policies, we will obtain a partial waiver of HIPAA authorization so that clinical staff may identify patients meeting eligibility criteria and introduce those individuals to a research coordinator embedded within the clinic. The research coordinator will provide patients with IRB-

approved advertisements describing the study. The research coordinator will also schedule an intake visit for interested patients, where patients will undergo the informed consent process (including full HIPAA authorization) in a private and confidential setting. We will also obtain a partial HIPAA waiver to allow undecided participants to provide us with contact information (i.e., name and phone number) so that we may contact them at a later date. The intake visit (which includes informed consent, randomization, assessment, and installing the smartphone app on participants' phones) will take approximately 1 hour.

Informed consent. The informed consent of each subject, using IRB-approved consent materials, will be obtained before that subject begins any study procedures. All subjects will be provided a written informed consent form at their intake session describing this study in language understandable to the study population. Research staff obtaining consent will review the consent document with subjects and thoroughly explain what the subjects need to know about the study, including study requirements, study risks and benefits. The consent discussion will occur at the intake session, although subjects will be given as much time as needed to make their decision, including rescheduling the intake appointment to another day. Participation privacy will be maintained and questions regarding participation will be answered. No coercion or undue influence will be used in the consent process. This consent form must be signed by the subject and the person obtaining the consent. The participant will receive a paper copy of the signed consent form, and the informed consent process will be documented in the research record.

Randomization. MAT clients with comorbid alcohol or marijuana use are placed on a slower progression from Phase I to Phase II (on-site administration every two weeks with 13 days of take-home medication) to reduce potential misuse of take-home Suboxone. Also, chronic pain is prevalent in this population, with 40% of our pilot participants reporting that pain has moderate or severe interference with daily activities (PROMIS Pain Interference Short form v4a). To address the possibility that chronic pain and/or comorbid use of alcohol or marijuana may influence treatment outcomes, randomization order to the OptiMAT (N=120) or Monitoring (N=120) arm will stratified by pain intensity (none/low vs. moderate/severe) and comorbid alcohol or marijuana use (yes vs. no). Assignment within each of the four strata (lower pain+no comorbidity; lower pain+comorbidity; higher pain+no comorbidity; higher pain+ comorbidity) will be determined at study initiation by 1:1 blocked randomization (block size n=4) using R software package blockrand which can be used to produce randomization lists and cards. This process ensures that every 4 consecutive group assignments contains randomly ordered assignments to 2 OptiMAT and 2 Monitoring arms. A separate randomization scheme will be developed for each study site, to ensure than each site has balanced recruitment into the two study arms. Group assignments will be written on notecards, sealed in envelopes, and placed in order within a shoebox. During Intake, after the participant has consented and determined to be eligible, the research coordinator will draw the next envelope in the queue to determine group assignment.

Additional smartphone app considerations. Participants in the smartphone app arm will be asked to use the app for 6 months. Participants will continue to have access to the app after their study participation ends, up to the 5-year period of NIH funding (tentatively ending fall 2026). If the DSMB find preliminary evidence that the OptiMAT arm leads to better outcomes than the Monitoring Only arm, then participants in the Monitoring Only arm will be invited to use the app and the end of this 6 month trial.

Retention. Since participants will also be patients with recurring appointments at CAST or the affiliated clinic, our research coordinator embedded in the clinic will have regular contact with participants continuing treatment. The research coordinator will also have contact information (i.e., phone number) for all participants, including those discontinuing treatment. The research coordinator will contact all participants monthly for Time-Line FollowBack assessments of opiate and other drug use, including participants discontinuing treatment. Clinical staff also collect primary contact information (i.e., friend, spouse, or relative) for its patients, and follows up on patients missing appointments to increase retention.

Compensation. Participants will receive at intake $60, or a BLU Grand M 8 GB smartphone (retail value $60). Participants will receive $10 for completing each of the 6 monthly phone call TLFB calendar assessments ($60 total). Participants who complete all 6 of the monthly phone calls will receive a $50 completion bonus, for a max total of $170. Participants in the fMRI substudy will receive $100 for the intake visit/first MRI, $100 for the month 2 MRI, and $100 for the month 4 MRI. Participants in the fMRI substudy will also receive ~$20 for completing the Money task each visit. ($300 + ~$60 = ~$360 total).

MRI sub-study. At the conclusion of the Intake visit, study staff will provide participants with information about the MRI sub-study and conduct a brief assessment to determine eligibility. Study staff will schedule an intake MRI visit for interested and potentially eligible participants. The MRI intake will last approximately 3 hours and consist of informed consent for the MRI sub-study, a SCID-5 clinical interview screening for comorbid psychiatric disorders and assessing severity of opioid use disorder, and additional assessments. BIRC research staff are trained to administer DSM-5 by Ms. Natalie Morris, our lead research assistant with over 15 years of experience administer these versions (and earlier versions) of structured clinical interviews. As per BIRC policy, each research staff is (a) given didactic instruction in DSM-5/MINI-KID interview administration, (b) practices the interview with Ms. Morris pretending to have one of several diagnosis, and (c) conducts 2-3 interviews under Ms. Morris's supervision, to ensure consistent interview fidelity across all participants.

Participants will then be scheduled for a MRI visit lasting approximately 2 hours. The MRI visit will be repeated at 2 months and 4 months. Participants will receive urinalysis screening for drugs of abuse and pregnancy (if applicable) at each MRI study visit.

Financial Interests and Intellectual Property. UAMS will trademark and copyright OptiMAT on behalf of Dr. James and the study team. Dr. James and the study team may have a future financial interest in OptiMAT, should OptiMAT be licensed for commercial use in future studies. For this study, OptiMAT will be available to participants at no cost. To ensure clinical trial study integrity, the staff statistician Xiaotong Han MS (a statistician in the department of Psychiatry who is blind to group membership and is not supervised by the PI or any faculty on this proposal) will conduct the statistical analysis of intervention effect. The DSMB will review and provide oversight to Mrs. Han's findings, including determinations of intervention-related adverse events.

Dissemination of Data

Results of this study may be used for presentations, posters, or publications. The publications will not contain any identifiable information that could be linked to a participant. Persuant to NIH/NIDA policy for transparency and rigorous experimental design (NOT-MH-14-004, NOT-DA-14-007), all published data will be de-identified and made publicly available through neuroimaging repositories such as the ENIGMA Addiction Working Group, INDI, or OpenFMRI. To promote open science, data infrastructure will follow the HCP universal BIDS format.

The study will be pre-registered at clinicaltrials.gov in accordance with NIH requirements.

Pilot study to evaluate OptiMAT interface

Before starting the RCT, we will recruit up to 10 patients receiving MAT for opioid use disorders to evaluate the OptiMAT app. These participants will attend a one-hour session to learn how to use the app. After two weeks, they will attend a brief individual focus group to give feedback about the app. (Please see attached instrument "OptiMAT Pilot Focus Group Questions".) These participants will not be enrolled in the RCT, and their data will thus not be included in any outcome analyses. Their focus group feedback will be given to the Enqbator app development team to improve and/or debug app features. Participants will be compensated $50 for their time ($25 at intake, $25 upon giving feedback after 2 weeks usage).

These pilot participants will also be invited to complete an MRI Intake Session and one MRI session. The Intake Session will only consist of instruments needed for the MRI task (i.e. MRI Safety Screening, attentional

bias cue word generator) and will not include other instruments (i.e. no SCID clinical interview, cognitive assessments, etc.). They will be compensated $50 total for their time (prorated), plus money won from the Money task (~$20). Their feedback will be used to improve the fMRI tasks for participants in the RCT. We will not use their fMRI data in any outcome analyses.

Literature Cited

1. Hasin, D. S., Aharonovich, E. & Greenstein, E. HealthCall for the smartphone: technology enhancement of brief intervention in HIV alcohol dependent patients. *Addict Sci Clin Pract* **9**, 5 (2014).
2. Thompson Jr., R., Aivadyan, C., Aharonovich, E. & Hasin, D. Smartphone application plus brief motivational intervention reduces substance use and sexual risk behaviors among homeless young adults: Results from a randomized controlled trial. *Psychology of Addictive Behaviors* (in press).
3. Dunlop, B. W. *et al.* Functional Connectivity of the Subcallosal Cingulate Cortex And Differential Outcomes to Treatment With Cognitive-Behavioral Therapy or Antidepressant Medication for Major Depressive Disorder. *Am J Psychiatry* **174**, 533–545 (2017).
4. Sinha, R., Catapano, D. & O'Malley, S. Stress-induced craving and stress response in cocaine dependent individuals. *Psychopharmacology (Berl.)* **142**, 343–351 (1999).
5. Sinha, R. Modeling stress and drug craving in the laboratory: implications for addiction treatment development. *Addiction Biology* **14**, 84–98 (2009).
6. Kilts, C. D., Gross, R. E., Ely, T. D. & Drexler, K. P. G. The neural correlates of cue-induced craving in cocaine-dependent women. *Am J Psychiatry* **161**, 233–241 (2004).
7. Kilts, C. D. *et al.* Individual Differences in Attentional Bias Associated with Cocaine Dependence Are Related to Varying Engagement of Neural Processing Networks. *Neuropsychopharmacology* **39**, 1135–1147 (2014).
8. Optseq Home Page. https://surfer.nmr.mgh.harvard.edu/optseq/.
9. Ekhtiari, H., Kuplicki, R., Pruthi, A. & Paulus, M. Methamphetamine and Opioid Cue Database (MOCD): Development and Validation. *Drug and Alcohol Dependence* **209**, 107941 (2020).
10. Knutson, B., Adams, C. M., Fong, G. W. & Hommer, D. Anticipation of increasing monetary reward selectively recruits nucleus accumbens. *J Neurosci* **21**, RC159 (2001).
11. Gustafson, D. H. *et al.* The effect of bundling medication-assisted treatment for opioid addiction with mHealth: study protocol for a randomized clinical trial. *Trials* **17**, 592 (2016).
12. Kennedy, A. P., Gross, R. E., Ely, T., Drexler, K. P. G. & Kilts, C. D. Clinical correlates of attentional bias to drug cues associated with cocaine dependence. *Am J Addict* **23**, 478–484 (2014).
13. Baron, R. M. & Kenny, D. A. The Moderator-Mediator Variable Distinction in Social Psychological Research: Conceptual, Strategic, and Statistical Considerations. 10.
14. Babor, T. F., Stenius, K. & Romelsjo, A. Alcohol and drug treatment systems in public health perspective: mediators and moderators of population effects. *Int J Methods Psychiatr Res* **17 Suppl 1**, S50-59 (2008).
15. Worley, M. J., Tate, S. R., Granholm, E. & Brown, S. A. Mediated and Moderated Effects of Neurocognitive Impairment on Outcomes of Treatment for Substance Dependence and Major Depression. *J Consult Clin Psychol* **82**, 418–428 (2014).
16. Dennis, M. L., Scott, C. K., Funk, R. R. & Nicholson, L. A Pilot Study to Examine the Feasibility and Potential Effectiveness of Using Smartphones to Provide Recovery Support for Adolescents. *Subst Abus* **36**, 486–492 (2015).
17. Kenny, D. A., Kashy, D. A. & Bolger, N. Data Analysis in Social Psychology. in *Handbook of social psychology* 233–265 (McGraw-Hill, 1988).
18. Tibshirani, R. Regression Shrinkage and Selection via the LASSO. *Journal of the Royal Statistical Society. Series B (Methodological)* **58**, 267–288.
19. Tibshirani, R. The lasso method for variable selection in the cox model. in *Statistics in Medicine* 385–395 (1997).

20. Sobell, L. C., Brown, J., Leo, G. I. & Sobell, M. B. The reliability of the Alcohol Timeline Followback when administered by telephone and by computer. *Drug & Alcohol Dependence* **42**, 49–54 (1996).
21. Robinson, S. M., Sobell, L. C., Sobell, M. B. & Leo, G. I. Reliability of the Timeline Followback for cocaine, cannabis, and cigarette use. *Psychol Addict Behav* **28**, 154–162 (2014).
22. Hoeppner, B. B., Stout, R. L., Jackson, K. M. & Barnett, N. P. How good is fine-grained Timeline Follow-back data? Comparing 30-day TLFB and repeated 7-day TLFB alcohol consumption reports on the person and daily level. *Addict Behav* **35**, 1138–1143 (2010).
23. Pedersen, E. R., Grow, J., Duncan, S., Neighbors, C. & Larimer, M. E. Concurrent Validity of an Online Version of the Timeline Followback Assessment. *Psychol Addict Behav* **26**, 672–677 (2012).
24. Rueger, S. Y., Trela, C. J., Palmeri, M. & King, A. C. Self-Administered Web-Based Timeline Followback Procedure for Drinking and Smoking Behaviors in Young Adults. *J Stud Alcohol Drugs* **73**, 829–833 (2012).
25. Garland, E. L., Froeliger, B. E., Passik, S. D. & Howard, M. O. Attentional bias for prescription opioid cues among opioid dependent chronic pain patients. *J Behav Med* **36**, 611–620 (2013).
26. Constantinou, N. *et al.* Attentional bias, inhibitory control and acute stress in current and former opiate addicts. *Drug and Alcohol Dependence* **109**, 220–225 (2010).
27. Browne, K. C., Wray, T. B., Stappenbeck, C. A., Krenek, M. & Simpson, T. L. Alcohol Consumption, Craving, and Craving Control Efforts Assessed Daily in the Context of Readiness to Change Among Individuals with Alcohol Dependence and PTSD. *Journal of Substance Abuse Treatment* **61**, 34–41 (2016).
28. Ferguson, S. G. & Shiffman, S. The relevance and treatment of cue-induced cravings in tobacco dependence. *Journal of Substance Abuse Treatment* **36**, 235–243 (2009).
29. Witkiewitz, K., Bowen, S. & Donovan, D. M. Moderating effects of a craving intervention on the relation between negative mood and heavy drinking following treatment for alcohol dependence. *J Consult Clin Psychol* **79**, 54–63 (2011).
30. Victor, T. A., Drevets, W. C., Misaki, M., Bodurka, J. & Savitz, J. Sex differences in neural responses to subliminal sad and happy faces in healthy individuals: Implications for depression. *J. Neurosci. Res.* **95**, 703–710 (2017).
31. Carlson, J. M., Aday, J. S. & Rubin, D. Temporal dynamics in attention bias: effects of sex differences, task timing parameters, and stimulus valence. *Cogn Emot* **33**, 1271–1276 (2019).
32. Pfabigan, D. M., Lamplmayr-Kragl, E., Pintzinger, N. M., Sailer, U. & Tran, U. S. Sex differences in event-related potentials and attentional biases to emotional facial stimuli. *Front Psychol* **5**, 1477 (2014).
33. Elton, A. *et al.* Childhood maltreatment is associated with a sex-dependent functional reorganization of a brain inhibitory control network: Childhood Trauma and Inhibitory Control. *Human Brain Mapping* **35**, 1654–1667 (2014).
34. Elton, A., Smitherman, S., Young, J. & Kilts, C. D. Effects of childhood maltreatment on the neural correlates of stress- and drug cue-induced cocaine craving. *Addict Biol* **20**, 820–831 (2015).
35. Martins, B. S., Cáceda, R., Cisler, J. M., Kilts, C. D. & James, G. A. The neural representation of the association between comorbid drug use disorders and childhood maltreatment. *Drug Alcohol Depend* **192**, 215–222 (2018).
36. Martins, B. S. The Crux of Addiction Comorbidity: fMRI analysis of how Childhood Trauma affects Network Functional Connectivity in the Comorbid Brain. (University of Arkansas for Medical Sciences, 2013).
37. Browning, M., Holmes, E. A., Murphy, S. E., Goodwin, G. M. & Harmer, C. J. Lateral Prefrontal Cortex Mediates the Cognitive Modification of Attentional Bias. *Biol Psychiatry* **67**, 919–925 (2010).
